# Pan-cancer exploration of oncogenic and clinical impacts revealed that HOXA9 is a diagnostic indicator of tumorigenesis

**DOI:** 10.1007/s10238-024-01389-x

**Published:** 2024-06-21

**Authors:** U. Sangeetha Shenoy, Dhanraj Salur Basavarajappa, Shama Prasada Kabekkodu, Raghu Radhakrishnan

**Affiliations:** 1 Department of Cell and Molecular Biology, Manipal School of Life Sciences, Manipal Academy of Higher Education, Manipal, Karnataka 576104 India; 2 Department of Oral Pathology, Manipal College of Dental Sciences, Manipal Academy of Higher Education, Manipal, Karnataka 576104 India; 3 Academic Unit of Oral and Maxillofacial Medicine and Pathology, School of Clinical Dentistry, University of Sheffield, Sheffield,, S10 2TA UK

**Keywords:** HOXA9, TCGA dataset, Cancer progression, Immune infiltration, Therapeutic drugs

## Abstract

Homeodomain transcription factor A9 (HOXA9) is a member of the HOX cluster family of transcription factors that are crucially involved in embryo implantation, morphogenesis, body axis development, and endothelial cell differentiation. Despite numerous reports on its aberrant expression in a few malignancies, the molecular and functional complexity of HOXA9 across cancers remains obscure. We aimed to analyze the dynamic role of *HOXA9* across cancers by identifying, analyzing, and understanding its multiple modes of regulation and functional implications and identifying possible therapeutic avenues. We conducted a comprehensive analysis to determine the role of *HOXA9* across cancers. This approach involved the integration of large-scale datasets from public repositories such as the Genomic Data Commons, specifically the Cancer Genome Atlas (GDC-TCGA), across 33 different cancer types. The multiple modes of *HOXA9* regulation by genetic and epigenetic factors were determined using online tools, which comprised experimentally validated observations. Furthermore, downstream pathways were identified by predicting the targets of HOXA9 and by performing functional enrichment analysis. We also assessed the clinical significance of *HOXA9* in terms of prognosis and stage stratification. This study evaluated the correlation between *HOXA9* and tumor-infiltrating molecules and discussed its association with therapeutically approved antineoplastic drugs. *HOXA9* was significantly upregulated in 9 tumors and downregulated in 2 cancers. The deregulation of *HOXA9* is primarily attributed to epigenetic factors, including promoter DNA methylation and noncoding RNAs (ncRNAs). The HOXA9 transcription factor interacts with PBX/MEIS cofactors and regulates multiple genes involved in cancer-associated EMT, autophagy, the cell cycle, metabolic pathways, Wnt signaling, TGF-β signaling, the AMPK pathway, PI3K/AKT signaling, and NF-κB signaling, thereby establishing control over downstream mechanisms. Differential expression in various clinical stages across cancers was shown to have prognostic significance and to be correlated with tumor-infiltrating immune molecules. The assessment of the correlation of *HOXA9* expression with approved antineoplastic drugs revealed that targeting HOXA9 could be the most reliable strategy for preventing cancer progression. HOXA9 is upregulated in the majority of malignancies and drives cancer progression by regulating multiple signaling mechanisms. Hence, HOXA9 could be a reliable diagnostic indicator and a potential therapeutic candidate for solid cancer types.

**Supplementary Information:**

The online version contains supplementary material available at 10.1007/s10238-024-01389-x.

## Introduction

Homeobox (HOX) genes are an evolutionarily conserved, unique set of genes embedded in four different HOX clusters that mainly function as regulators of morphogenesis and body axis specifications [1, 2]. All 39 HOX genes function as homeodomain transcription factors to regulate diverse biological targets. *HOXA9*, a member of the HOXA cluster, plays a crucial role in various biological processes, including body axis specification, morphogenesis, endothelial cell proliferation, and embryo implantation [3–6]. Its involvement is not only limited to normal developmental processes but also extends to its aberrant expression in both solid cancers and hematological malignancies [7]. As a HOX protein, it has a unique “DNA binding homeodomain”, which helps in binding to target genes with HOXA9-specific consensus sequences. As a transcription factor, HOXA9 exerts its influence by regulating a diverse set of targets that contribute to cancer-related events, further emphasizing its significance in the context of oncology. Hence, the dysregulation of *HOXA9* expression underscores its potential as a key player in the molecular mechanisms associated with cancer progression. To the best of our knowledge, its functional role in cancer progression, its association with immune subtypes, and its clinical importance across cancers have not yet been explored.

As published previously, HOXA9 plays a profound role in regulating cancer-associated biological events when aberrantly expressed in cancer [8]. In most solid cancer types, aberrant expression is primarily attributed to epigenetic factors, while in hematological malignancies, *HOXA9* gene fusion is more frequent [7, 9]. Consequently, downstream pathways contribute to the progressive development of tumors. It functions either as a tumor promoter or as a tumor suppressor, depending on the tissue-specific cancer type. Researchers have shown that HOXA9 induces cell proliferation, stemness, angiogenesis, invasion, and metastasis in several cancer types, including colorectal cancer (CRC)[10–12], pancreatic cancer (PC)[13], prostate cancer (PCa)[14], osteosarcoma[15], and glioma (GBM)[16–18], where it also confers resistance to drugs and promotes cancer recurrence. In vitro studies have shown that *HOXA9* functions as a tumor suppressor in cutaneous squamous cell carcinoma (CSCC) [19, 20], cervical cancer (CC) [21], breast cancer (BC) [22, 23], non-small cell lung cancer (NSCLC) [24–27], epithelial ovarian cancer (EOC) [28], high-grade noninvasive bladder cancer (HGNOC) [29], uveal melanoma [30] and lung adenocarcinoma (LUAD) [31]. Nevertheless, the molecular mechanism underlying its aberrant expression and its functional implications in all these cancer types have not been elucidated.

In the present study, we systematically analyzed the expression status of *HOXA9* in 33 different cancer types using datasets from GDC-TCGA. Multiple modes of *HOXA9* regulation have been determined through a comprehensive analysis of genetic and epigenetic data, which revealed that HOXA9 is correlated with gene expression status. To assess the functional role of this gene as a transcription factor, we predicted diverse biological targets, which were subsequently subjected to functional enrichment analysis. In addition to investigating the clinical significance of *HOXA9* in prognosis and cancer stages, we identified a potential association between *HOXA9* expression and various immune subtypes across cancers, enabling us to correlate *HOXA9* expression with individual immune cell types. Hence, this study was conducted to explore the significant role of *HOXA9* in multiple cancer types, with a particular focus on its involvement in immunological response, thereby offering new insights into anticancer therapy.

## Materials and methods

### Data acquisition, processing, and cross-validation

The gene expression data for *HOXA9* from 50 different normal tissues were obtained from the consensus dataset in the Human Protein Atlas (HPA) (https://www.proteinatlas.org/) [32]. The differential expression of *HOXA9* between solid tissue normal and primary tumor samples in multiple cancer types was visualized as a box plot in the TIMER2.0 database under the cancer exploration ‘Gene DE’ module (http://timer.cistrome.org/) [33]. Additionally, it consists of modules that systematically provide clinical, immunological, and genomic features of a specific gene in the pan-cancer TCGA cohort. The *HOXA9* gene expression in 33 individual cancer types was retrieved from RNA-seq data of GDC-TCGA datasets and reposited in the UCSC Xena browser, where the values are represented in log2 (fpkm-uq + 1) (https://xena.ucsc.edu/) [34]. The differential expression in each cancer type was further verified by performing the Wilcoxon test, and the data are represented in box plots using SRplot (https://www.bioinformatics.com.cn/srplot) [35]. A pvalue < 0.05 was considered to indicate statistical significance. Similarly, *HOXA9* expression in cancer cell lines was retrieved using the Cancer Cell Line Encyclopedia (CCLE) portal (https://sites.broadinstitute.org/ccle/) [36]. The analyzed data were further cross-validated with samples of Affymetrix U133A (GPL96) and U133Plus2 (GPL570) microarray platforms conjugated in another database, namely GENT2, which collects data from the National Centre for Biotechnology Information-Gene Expression Omnibus (NCBI-GEO) public datasets and processes them using the Bioconductor package with the MAS5 algorithm [37]. Moreover, we evaluated the protein expression of HOXA9 in different tumor types with respect to that in normal tissues from immunohistochemistry (IHC) data in the HPA dataset.

### Genetic and epigenetic alteration analysis

Mutations at the *HOXA9* locus in TCGA-cancer types were retrieved from the TIMER2.0 database. This database provides the percentage of samples with *HOXA9* mutations in TCGA cancer types in the form of a bar plot. Furthermore, single-nucleotide variations (SNVs), including missense mutations, nonsense mutations, and frameshift deletions, represented the percent frequency of mutations in tumor samples. Copy number variations (CNVs), such as homologous-heterologous amplifications and deletions, were determined using the GSCA (Gene Set Cancer Analysis) database (http://bioinfo.life.hust.edu.cn/GSCA/#/) [38]. Using the same database, the correlation between CNVs and the expression of *HOXA9* was determined. The epigenetic alterations of *HOXA9* were further examined using TCGA datasets. Differential patterns of *HOXA9* promoter DNA methylation in tumor samples compared with normal samples and their correlation with gene expression were determined by retrieving and analyzing the methylation data in the same GDC-TCGA datasets from the UCSC Xena browser, which was utilized for gene expression analysis. Furthermore, we retrieved the miRNAs sponging *HOXA9* from 3 different databases, namely miRTarBase [39], miRBase [40], and starBase v2.0 [41], which provide experimentally validated miRNA‒mRNA sponges. After retrieving all the miRNAs from the three different databases, overlapping analysis was performed using the Venny 2.1 tool (https://bioinfogp.cnb.csic.es/tools/venny/). The gene-miRNA interaction network was plotted using Cytoscape 3.10.1 (https://cytoscape.org/release_notes_3_10_1.html) [42]. The correlations between these 12 miRNAs and *HOXA9* expression in the pan-cancer TCGA dataset were retrieved from starBase v2.0.

### HOXA9 coexpression and construction of a protein–protein interaction network (PPIN)

Furthermore, *HOXA9* coexpression analysis was performed using the GeneMANIA [43] and BioGRID [44] databases. Furthermore, the PPIN was generated using the STRING tool (https://string-db.org/) with a confidence score of 0.4 [45]. Common molecules from these three databases were identified, and their degree of correlation with *HOXA9* in TCGA cancer types was determined using the ‘Gene Correlation module’ in the TIMER2.0 database.

### HOXA9 target prediction and functional enrichment analysis

Computational tools, namely the Gene Transcription Regulation Database (GTRD) (http://gtrd.biouml.org) [46] TFlink (https://tflink.net/) [47], were used to determine the experimentally validated targets of the HOXA9 transcription factor and upstream molecules. The GTRD database identifies the transcription factor-binding sites validated by chromatin immunoprecipitation (ChIP) experiments; these sites were collected from public repositories, namely GEO, ENCODE, the Sequence Read Archive (SRA), and the literature. The TFlink database offers highly accurate and experimentally proven transcription factor-target gene interactions for six organisms, including humans. HOXA9 targets were subjected to functional enrichment analysis using the ShinyGO tool (http://ge-lab.org/go/) [48]. The enrichment fold changes, along with the FDRs and p values of all the pathways, were downloaded, and graphs were generated using SRplot.

### The role of HOXA9 in clinical staging and prognosis

The differential expression of *HOXA9* in cancer stages (stage I, stage II, stage III, and stage IV) was determined using the same GDC-TCGA datasets from the UCSC Xena browser. One-way ANOVA was performed to determine the statistical significance, and the graphs were generated by using GraphPad Prism 8. Furthermore, the prognostic significance of the *HOXA9* gene in TCGA cancer datasets was determined by conducting survival analysis using UALCAN [49] and KMplotter (https://kmplot.com/analysis/) [50]. The overall survival of the patients stratified by stage was also determined.

### Correlations of HOXA9 expression with different immune subtypes, immune cell infiltration, and drug-gene interactions

The associations between *HOXA9* expression and different immune subtypes of cancer were determined using the TISIDB database (http://cis.hku.hk/TISIDB) [51], which is a well-known repository for information on tumor–immune interactions. Next, we utilized the TIMER 2.0 tool to determine the correlation between *HOXA9* expression and major tumor-infiltrating cells, including macrophages, natural killer (NK) cells, CD4^+^/CD8^+^ T cells, B cells, and dendritic cells. The raw data were used to construct a heatmap showing the correlation with *HOXA9* in TCGA cancer types using SRplot. Accordingly, statistically significant values were represented as asterisks on the heatmap. Furthermore, we explored the GSCA database, which retrieves drug-gene correlation data from the GDSC and CTRP portals. The correlation between the use of therapeutically approved antineoplastic drugs and the expression of *HOXA9* has been represented in the form of a bubble plot. The graphs were generated by using SRplot [35] and analyzed by GraphPad Prism 8.

**Fig. 1:**
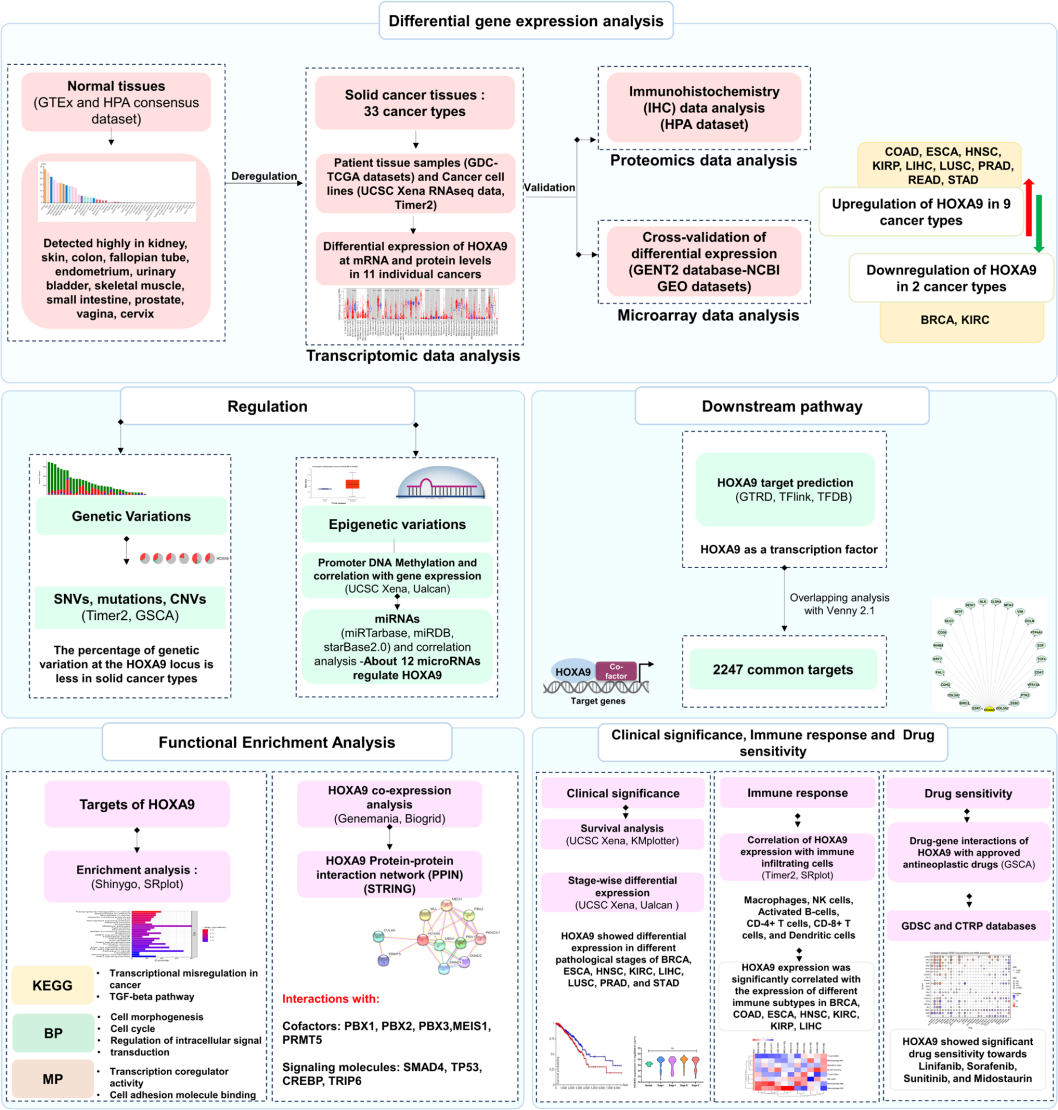
Workflow of in silico analysis performed in the present study

The methodology and results of the analysis are summarized in Fig. 1.

## Results

### HOXA9 is differentially expressed across cancers

**Fig. 2:**
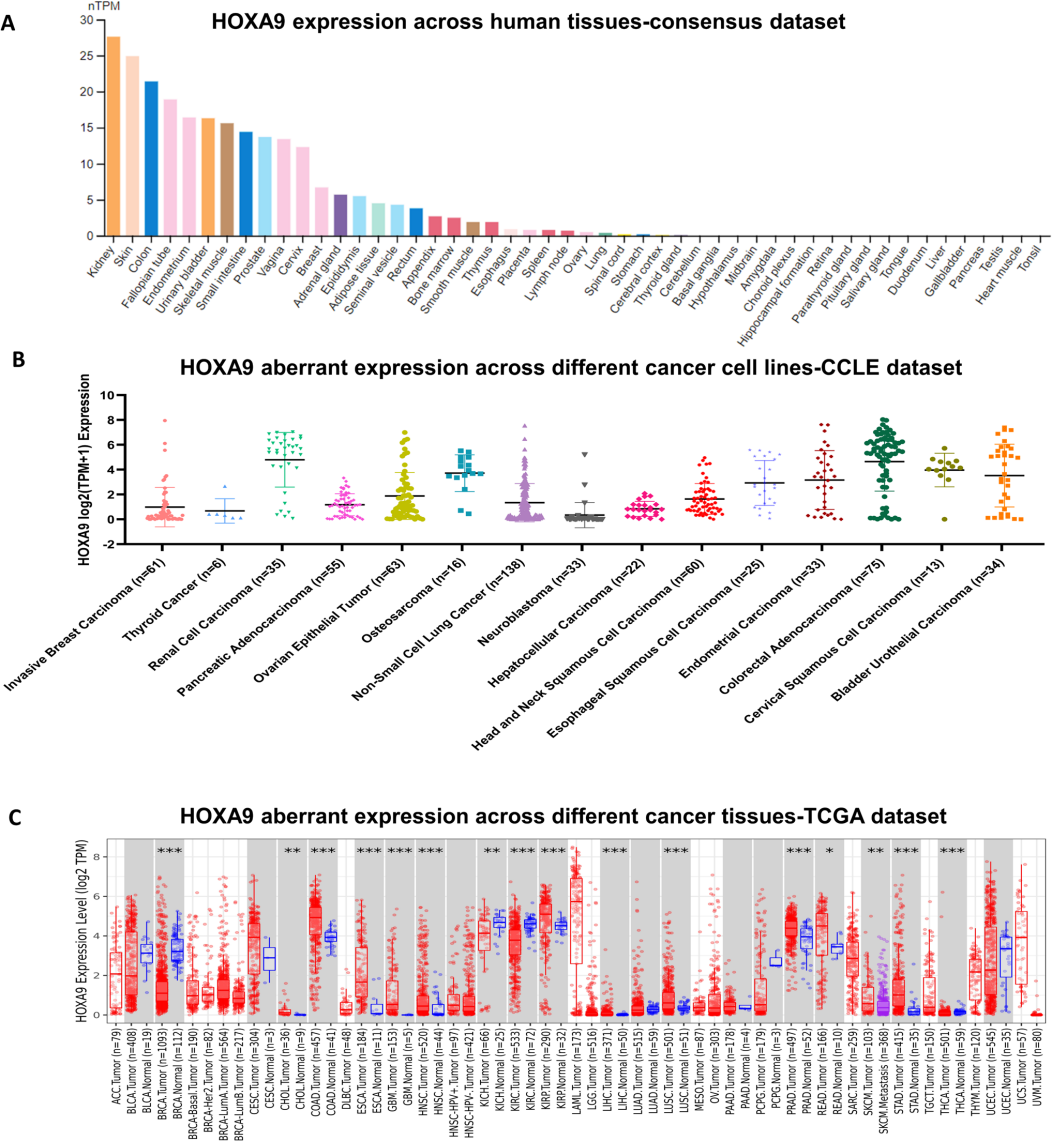
Gene expression analysis of *HOXA9*: **A** Expression of *HOXA9* in human tissues retrieved from a consensus dataset in the HPA database. **B** Expression of *HOXA9* across different cancer cell lines retrieved from the CCLE database. **C** Expression of *HOXA9* across different cancer tissues visualized by the TIMER 2.0 database

**Fig. 3:**
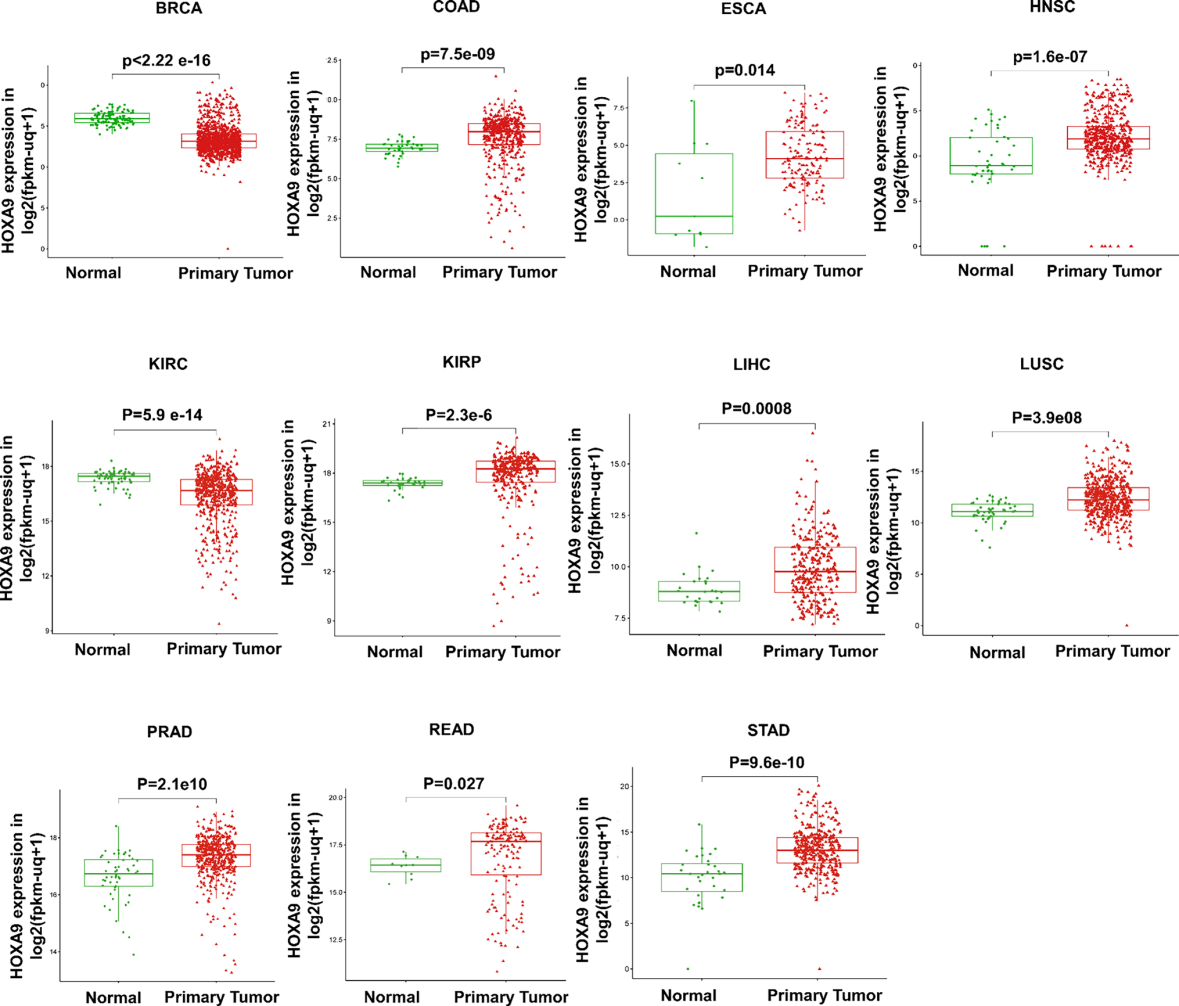
*HOXA9* gene expression in the TCGA RNA-Seq dataset retrieved from the UCSC Xena browser: Increased expression of *HOXA9* in COAD, ESCA, HNSC, KIRP, LIHC, LUSC, PRAD, READ, and STAD was observed in primary tumors compared to that in normal solid tissue. Downregulation of *HOXA9* expression was observed in BRCA and KIRP primary tumor tissues compared to normal solid tissue

By analyzing gene expression using consensus data from the HPA and GTEx datasets, we discovered that *HOXA9* is highly expressed in the kidney and skin under normal physiological conditions (Fig. 2A). Subsequently, we determined the expression of *HOXA9* in diverse cell lines associated with each cancer type retrieved from the CCLE portal (Fig. 2B). Initially, we checked the expression status of *HOXA9* in TCGA cancer types using the TIMER 2.0 database (Fig. 2C). Notably, *HOXA9* was aberrantly expressed in 11 out of 33 GDC-TCGA cancer types. *HOXA9* mRNA was highly elevated in the tissues of 9 solid cancers and downregulated in 2 of the cancer types compared to normal tissues. Tumor tissues of colon adenocarcinoma (COAD), esophageal carcinoma (ESCA), head and neck cancer (HNSC), kidney renal papillary cell carcinoma (KIRP), liver hepatocellular carcinoma (LIHC), lung squamous cell carcinoma (LUSC), prostate adenocarcinoma (PRAD), rectal adenocarcinoma (READ), and stomach adenocarcinoma (STAD) exhibited increased expression of *HOXA9* compared to that of the solid normal samples (*p* value < 0.05) (Fig. 3). However, *HOXA9* expression was significantly downregulated in breast invasive carcinoma (BRCA) and kidney renal clear cell carcinoma (KIRC) (*p* value < 0.05) (Fig. 3). Furthermore, information on HOXA9 protein expression was available for only a few of the cancer types analyzed using the HPA database and is provided in Supplementary Fig. 1.

The GPL570 dataset showed differential gene expression in tumor tissues of the breast, colon, esophagus, head and neck, kidney, liver, lung, prostate, and stomach. The GPL96 dataset showed differential *HOXA9* expression in the breast, colon, esophagus, kidney, lung, and stomach (Supplementary Fig. 2). These findings suggest that *HOXA9* may play a role in promoting carcinogenesis in multiple tumor types. Therefore, additional research is necessary for its clinical significance.

### Regulation of HOXA9 across cancers

**Fig. 4:**
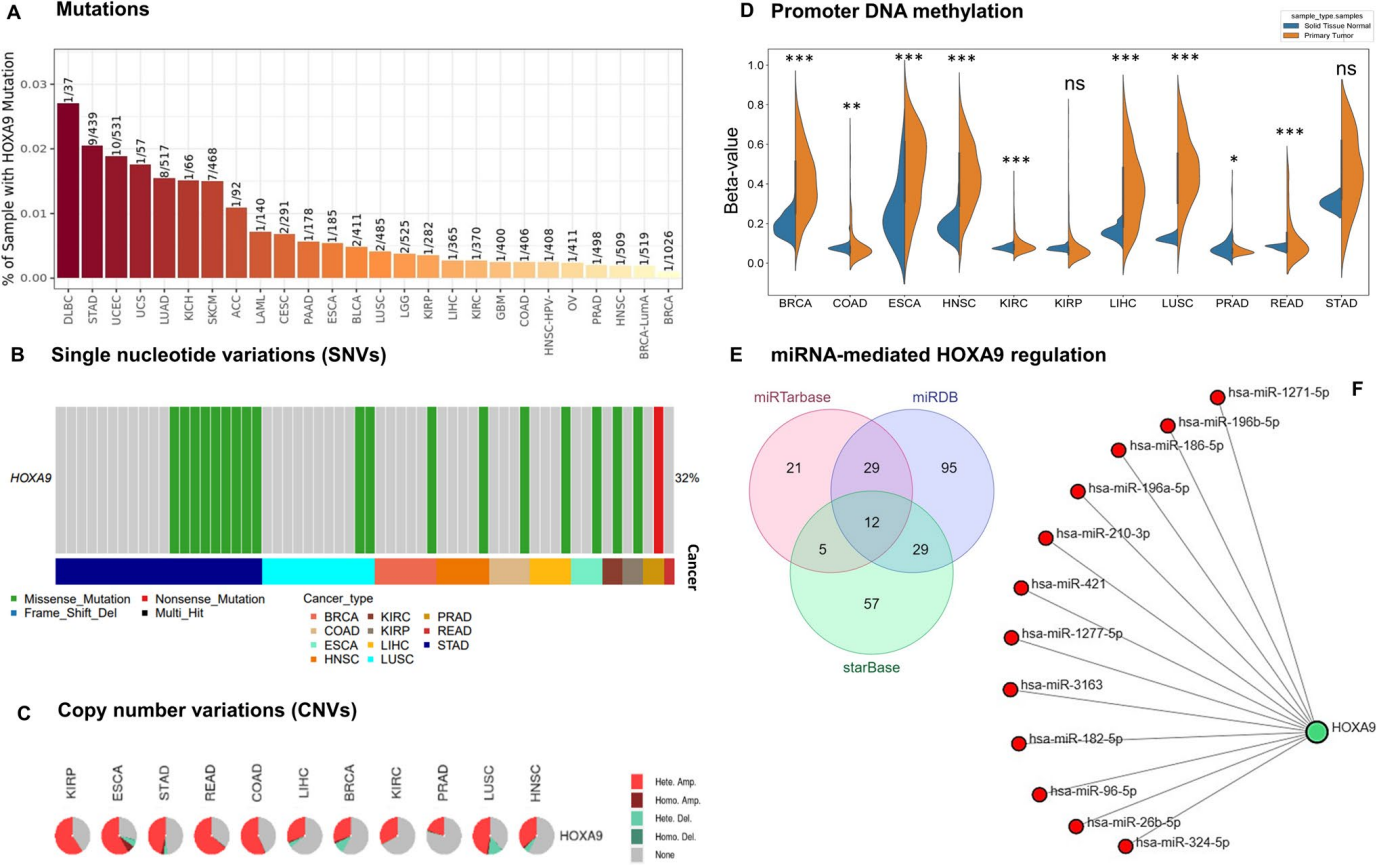
Genetic and epigenetic variation analysis of the *HOXA9* gene: **A** Percentage of mutations at the *HOXA9* locus visualized by the Timer2.0 database. **B**, **C** Types of SNVs and CNVs observed at the *HOXA9* locus in different cancer types visualized by the GSCA database. **D** Promoter DNA methylation of the *HOXA9* promoter was analyzed using TCGA datasets from the UCSC Xena browser and plotted using an SR plot. **E** Overlapping analysis of miRNAs regulating *HOXA9* using 3 different databases and the Venny 2.1 tool. F. Network of 12 miRNAs regulating *HOXA9*, generated using Cytoscape 3.10.1

Among the 11 TCGA cancer types, *HOXA9* exhibited the highest mutation frequency, with 9% in STAD, 2% in LUSC, and 1% in the remaining tumors. Despite missense mutations frequently occurring SNVs in almost all cancer types, the overall mutation rate across cancers was only 32% (Fig. 4A, B), indicating that other factors might regulate the expression of *HOXA9*. Subsequently, we checked whether the upregulation of *HOXA9* in 9 TCGA cancer types was attributed to CNVs at this locus. Interestingly, the *HOXA9* locus exhibited heterozygous amplification in more than 50% of patients across the 6 TCGA cancer types, with READ (64.24%) and ESCA (65.21%) showing the highest percentages (Fig. 4C). When we correlated CNVs with the expression of *HOXA9*, we found a strong positive correlation for KIRP (Spearman R-coefficient 0.513 and *p* value 7.4E−18) (Supplementary Table 1).

Then, we investigated the potential association between the upregulation of *HOXA9* expression and epigenetic variation by examining the promoter DNA methylation status across all 11 TCGA cancer types using the same GDC-TCGA datasets employed for gene expression analysis. Among the 11 TCGA cancer types, we found a significant difference in promoter DNA methylation in 9 cancer types (*p* value < 0.05) (Fig. 4D). Correlation analysis revealed that the reduced expression of *HOXA9* in BRCA and KIRC patients resulted from promoter DNA hypermethylation, while the elevated expression in PRAD patients was attributed to promoter DNA hypomethylation (Supplementary Table 2). Our study revealed that the upregulation of *HOXA9* in KIRP could mainly be due to CNVs because no differential methylation was detected between normal and primary tumor samples. However, there was no significant correlation between *HOXA9* expression and methylation in LUSC, and a weak correlation was observed in HNSC and LIHC (Supplementary Table 2), suggesting the role of other epigenetic factors. Despite being upregulated in HNSC, LIHC, COAD, ESCA, and READ primary tumors, *HOXA9* showed hypermethylation compared to that in normal solid tissue. This paradoxical association indicated complex and context-dependent regulation and has recently been reported in a few cancer types [52]. According to the correlation analysis performed in the present study, an inverse correlation was observed between expression and methylation in these cancer types, suggesting a strong need for experimental validation to pinpoint the precise promoter and its methylation status governing the expression in each of the cancer types. Analyzing the deposition pattern of histone marks on promoter regions could also provide valuable insights into their regulation.

We further performed a thorough analysis of three different databases with experimentally validated miRNA‒target gene interactions and identified 12 miRNAs as upstream epigenetic regulators of *HOXA9* that play a role in the regulation of its gene expression, namely hsa-miR-1271-5p, hsa-miR-1277-5p, hsa-miR-182-5p, hsa-miR-186-5p, hsa-miR-210-3p, hsa-miR-26b-5p, hsa-miR-3163, hsa-miR-324-5p, hsa-miR-421 and hsa-miR-96-5p. Notably, two HOX cluster-embedded miRNAs, namely hsa-miR-196a-5p and hsa-miR-196b-5p, were also found. The results of the correlation analysis of miRNA gene expression are summarized in Supplementary Table 3. However, Table 1 summarizes the malignancies in which these miRNAs showed an inverse correlation with *HOXA9*, which might be one of the possible driving factors for *HOXA9* regulation. Hence, experimental investigation is essential to understand the underlying driving epigenetic mechanism behind the aberrant expression of *HOXA9*.

**Table 1:** In silico prediction of miRNAs and their inverse correlation with *HOXA9* expression in pan-cancer

SI. no	miRNAs inversely correlate with HOXA9	Cancer types	Coefficient-*R*	*P* value
1	hsa-miR-1271-5p	Prostate adenocarcinoma	− 0.113	1.22E− 02
		Rectum adenocarcinoma	− 0.247	1.56E− 03
2	hsa-miR-1277-5p	Kidney renal clear cell carcinoma	− 0.109	1.32E− 02
3	hsa-miR-182-5p	Breast invasive carcinoma	− 0.105	5.25E− 04
4	hsa-miR-186-5p	Breast invasive carcinoma	− 0.081	7.84E− 03
		Kidney renal papillary cell carcinoma	− 0.2	6.21E− 04
		Rectum adenocarcinoma	− 0.16	4.31E− 02
5	hsa-miR-196a-5p	None	–	–
6	hsa-miR-196b-5p	None	–	–
7	hsa-miR-210-3p	Colon adenocarcinoma	− 0.094	4.55E− 02
		Breast invasive carcinoma	− 0.17	1.61E− 08
8	hsa-miR-26b-5p	Kidney renal papillary cell carcinoma	− 0.123	3.74E− 02
9	hsa-miR-3163	None	–	–
10	hsa-miR-324-5p	Breast invasive carcinoma	− 0.147	1.19E− 06
11	hsa-miR-421	Prostate adenocarcinoma	− 0.092	4.11E− 02
		Colon adenocarcinoma	− 0.099	3.55E− 02
12	hsa-miR-96-5p	Breast invasive carcinoma	− 0.142	2.75E− 06

### The HOXA9 transcription factor interacts with cofactors to strengthen DNA binding

**Fig. 5:**
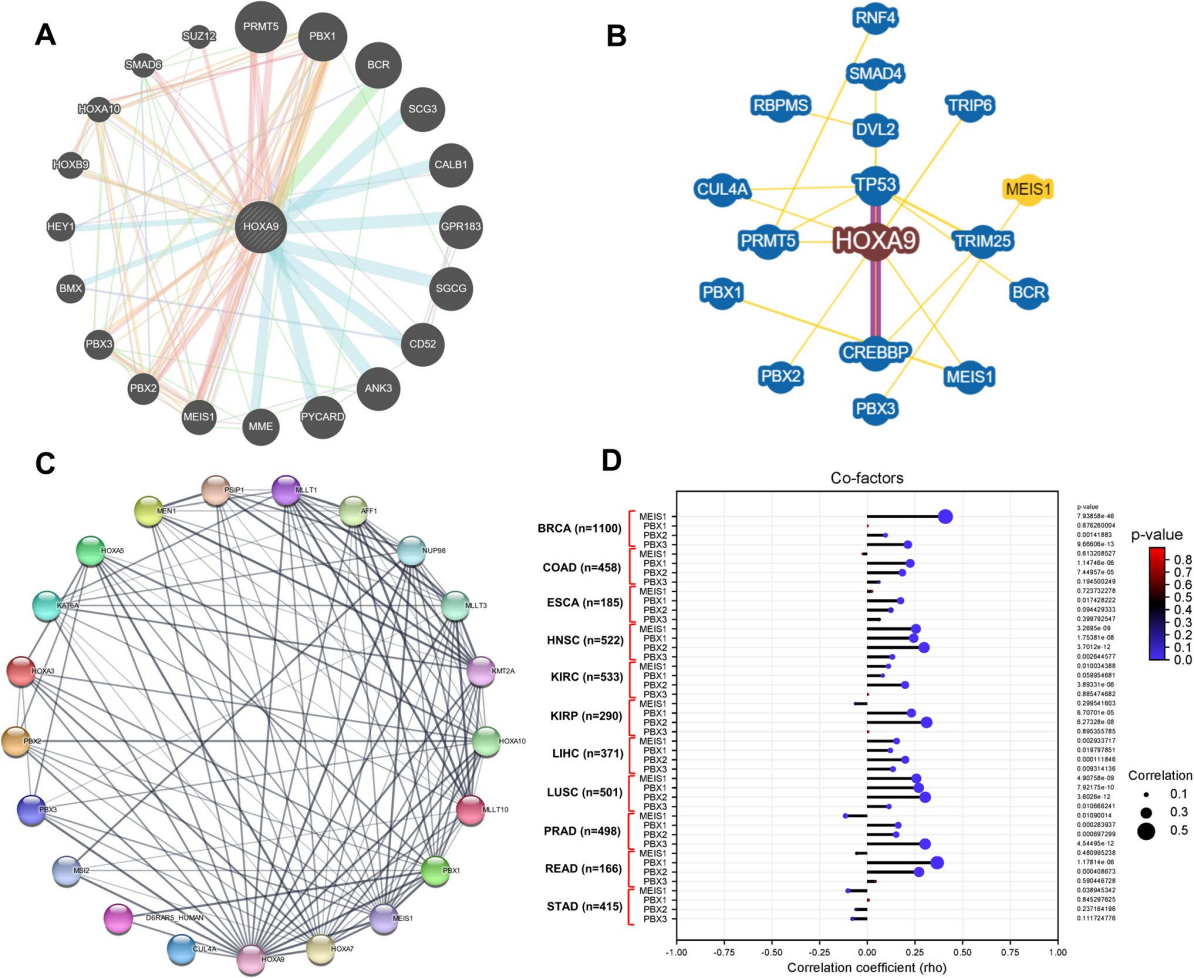
Coexpression analysis of HOXA9 using the **A** GeneMANIA, **B** Biogrid and **C** STRING databases. **D** Lollipop plot showing the correlation of HOXA9 with PBX1, PBX2, PBX3 and MEIS1 in different cancer types

Coexpression analysis of the coexpressed genes using GeneMANIA and BioGRID revealed several interacting factors (Fig. 5A, B). Our overlapping analysis revealed that *HOXA9* is primarily coexpressed with five genes: *MEIS1*, *PBX1*, *PBX2*, *PBX3*, *PRMT5,* and *PBX3*. Additionally, the interaction of the HOXA9 protein with the cofactors PBX1, PBX2, PBX3, and MEIS1 was observed and visualized in the PPIN (*p* value < 1.0e−16) (Fig. 5C). It was also shown to interact with other crucial regulators, namely CUL4A, D6RAR5, HOXA10, HOXA3, HOXA5, HOXA7, MLLT1, KAT6A, MSI2, MLLT10, NUP98, MEN1, PSIP1, MLLT3 and KMT2A. Analysis of the *HOXA9* gene revealed a positive correlation with PBX1, PBX2, PBX3, and MEIS1 in all cancer types except for STAD among the 11 studied genes, suggesting enhanced binding of HOXA9 to the promoter region of target genes (Fig. 5D).

### The HOXA9 transcription factor (TF) regulates important signaling molecules associated with *cancer*-related biological events

The ChIP-seq data derived from the TFlink database revealed the list of TFs that regulate *HOXA9* expression, and the targets regulated by the HOXA9 TF (Supplementary Table 4). However, our downstream target analysis revealed that HOXA9 functions as a transcription factor for 2247 genes (Supplementary Table 4). Angiogenic markers such as *GRB2, MAPK8, FGFR1, PI3KCA*, and *HIF1A,* as well as Wnt/β-catenin pathway markers such as *CREBBP, MYC, and NLK*, were identified as targets of HOXA9. Other targets were found to be involved in TGF-β signaling, PI3K signaling, NF-κB signaling, and VEGF signaling, all of which are associated with cancer progression. Notably, we identified the following EMT markers as *HOXA9* targets: *ESR1, BIRC3, COL5A1, CDH2, FHL1, KRT7, INHBA, CD36, DLG1, MITF, DESI1, NLK, CLDN4, MTA3, COL5A2, VIM, OCLN, PTP4A1, EGF, TCF4, CD47, VPS13A, PTK2* and *ZEB2*.

**Fig. 6:**
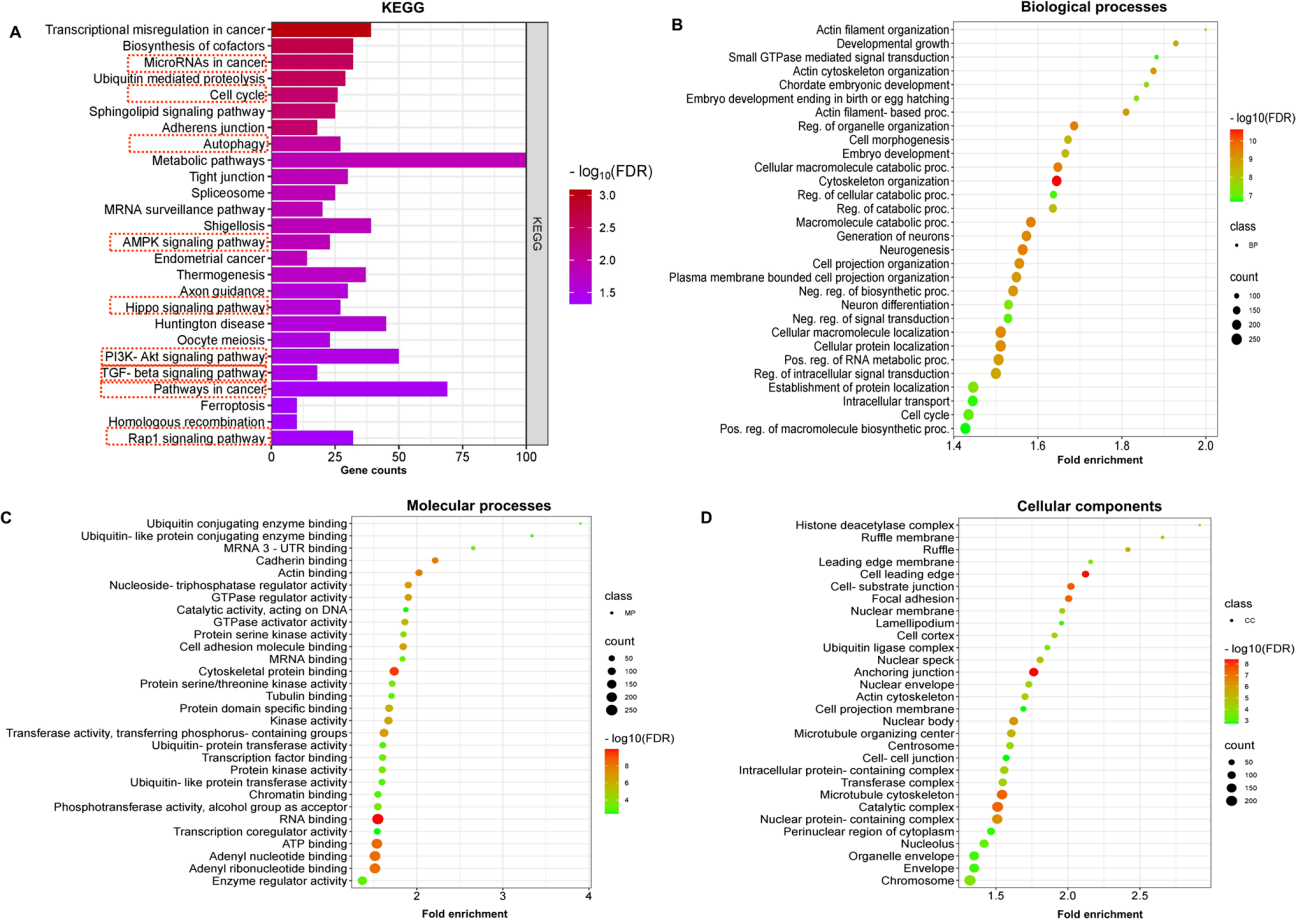
Functional enrichment analysis of HOXA9 using the ShinyGO tool. **A** KEGG pathway analysis. **B** Biological processes. **C** Molecular processes. **D** Cellular components

All 2247 targets of HOXA9 were subjected to enrichment analysis to assess their biological significance in cancer progression. KEGG pathway analysis revealed that the targets were enriched in autophagy, the cell cycle, metabolic pathways, the AMPK pathway, Hippo signaling, PI3K/AKT signaling, TGF-β signaling, Rap1 signaling, and transcriptional misregulation in cancer (Fig. 6A). Notably, the pathways represented by these targets were reported to be significantly involved in acquiring cancer hallmarks, indicating the ability of HOXA9 to regulate downstream pathways to promote cancer progression. The top biological process (BP) terms included actin filament organization, cell morphogenesis, cell cycle, negative regulation of signal transduction, and regulation of intracellular transduction (Fig. 6B). In addition, targets with the greatest enrichment in molecular processes (MPs), such as cadherin binding, GTPase regulator activity, protein serine-kinase activity, cell adhesion molecule binding, protein domain-specific binding, transcription factor binding, and transcription coregulator activity, were the most enriched (Fig. 6C). These targets were particularly localized in the cell‒cell junction, nuclear membrane, perinuclear region of the cytoplasm, and microtubule cytoskeleton (Fig. 6D). All the GO terms associated with HOXA9 targets are represented in the form of bubble plots in Fig. 6.

### Clinical significance of HOXA9 across cancers

The prognostic significance of *HOXA9* in GDC-TCGA datasets was determined by using the UALCAN database, which analyzes the correlation between the *HOXA9* gene and the prognosis of patient samples through follow-up studies. However, there was no significant difference between the expression of *HOXA9* and the prognosis of patients with any of the cancer types (Supplementary Fig. 3).

**Fig. 7:**
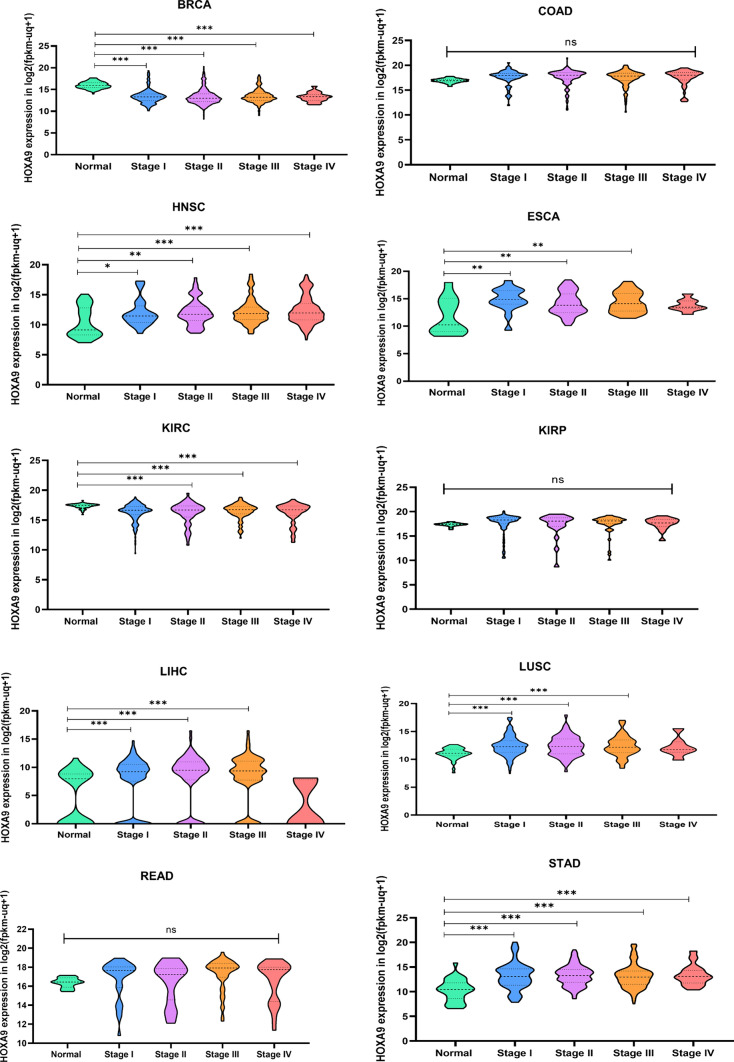
Expression of *HOXA9* in different clinical stages of TCGA cancers: Raw data retrieved from the UCSC Xena RNA-seq dataset were analyzed and plotted using GraphPad Prism 8

To evaluate the clinical utility of HOXA9 in stage stratification, we analyzed gene expression data across different clinical stages (stages I-IV) in 11 cancer types and revealed significant differential expression in BRCA, HNSC, ESCA, KIRC, LIHC, LUSC, and STAD (p value < 0.05), but not in COAD, KIRP, READ (not reported), or PRAD (Fig. 7). Assessing the expression of *HOXA9* in the different clinical stages might aid in preventing cancer progression. Hence, we performed survival analysis for patients stratified by stage I to IV disease. The prognostic significance was observed only for KIRC and KIRP in the 11 cancer types that we studied. Interestingly, over time, we observed better survival in KIRC patients with downregulated *HOXA9* expression and KIRP with upregulated *HOXA9* expression (*p* value < 0.05) (Supplementary Fig. 4). The upregulation (downregulation) of genes associated with improved survival outcomes was attributed to activation of the antitumor immune response [53, 54]. There could also be several possible reasons for this contradictory observation beyond the direct immune response, which has recently been reported in solid malignancies [55, 56]. Hence, this observation prompted us to investigate the underlying mechanism of the immune response.

### HOXA9 expression is correlated with immune cell infiltration across cancers

**Fig. 8:**
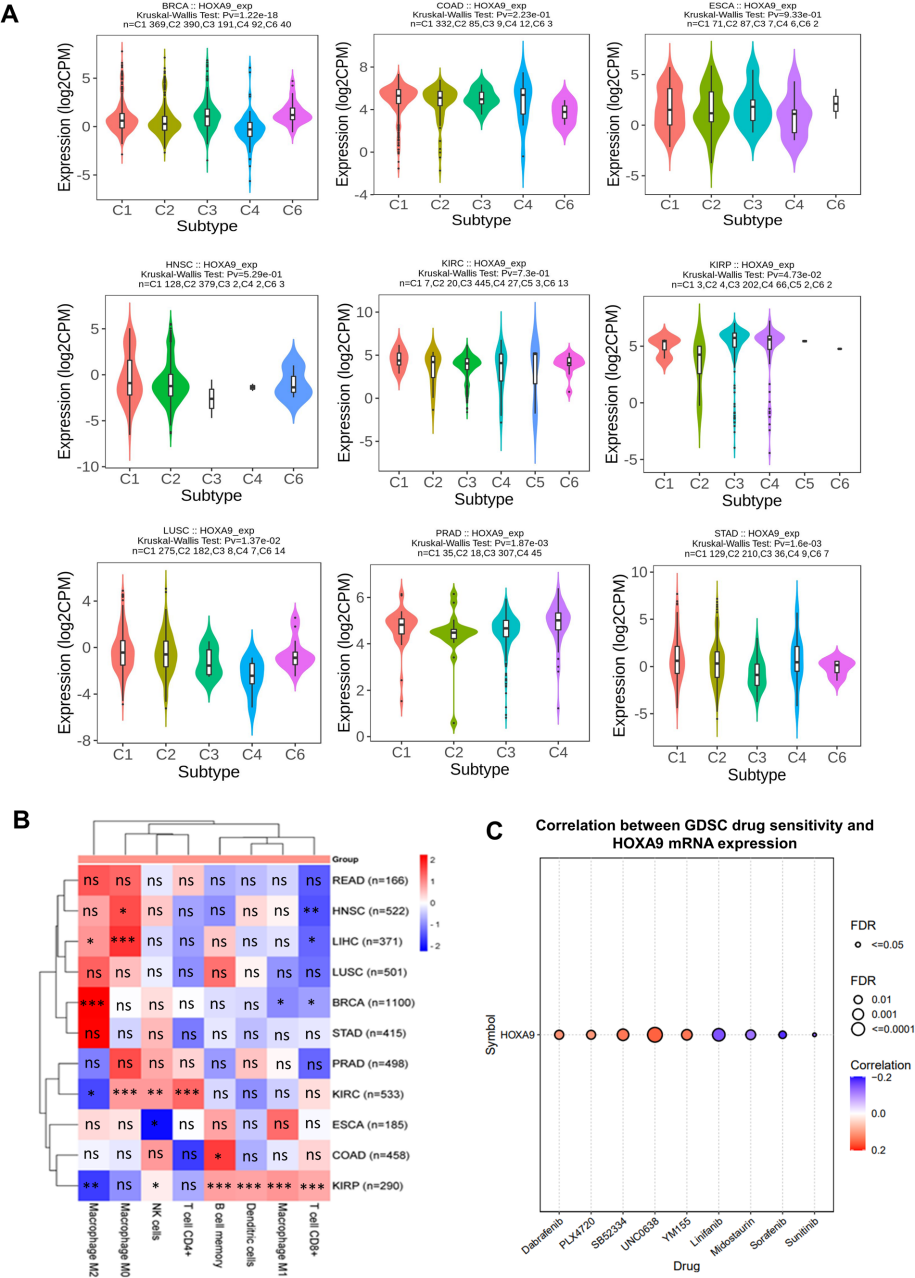
HOXA9 and immune interactions: **A** Expression of *HOXA9* in different immune subtypes visualized in the TISIDB database. **B** Heatmap showing the correlation between *HOXA9* expression and immune cells in different cancer types retrieved from the TIMER 2.0 database. **C** Bubble plot showing the correlation between *HOXA9* expression and sensitivity to the antineoplastic drugs from the GSCA database

*HOXA9* expression in various immune subtypes of each cancer type was assessed using the TISIDB database, categorizing immune subtypes into six groups based on the TISIDB algorithm: wound healing (C1), IFN-gamma dominant (C2), inflammatory (C3), lymphocyte depleted (C4), immunologically quiet (C5), and TGF-β dominant (C6). The results indicated that *HOXA9* exhibited differential expression in the immune subtypes BRCA, KIRP, LUSC, PRAD, and STAD (Fig. 8A).

Building on the findings of *HOXA9* expression with respect to immune subtypes, we conducted an extensive analysis to investigate its association with various immune cells, including CD4+ T cells, CD8+ T cells, B cells, dendritic cells, macrophages, and natural killer (NK) cells, in 11 TCGA cancer types. Our analysis using the TIMER2.0 database revealed that the overexpression of *HOXA9* was accompanied by the infiltration of immune cells (Fig. 8B). In particular, in KIRP, high expression of *HOXA9* was significantly positively correlated with the infiltration of CD8+ T cells, B-memory cells, dendritic cells, NK cells and M1 macrophages, which are all actively involved in promoting antitumor activity. In contrast, there was a negative correlation between *HOXA9* expression and protumorigenic M2 macrophages in KIRC. This finding strengthens our previous observation of HOXA9-mediated better survival outcomes in advanced stages of KIRC and KIRP, which might be mainly due to the involvement of HOXA9 in triggering immune response pathways and inducing immune cell activation and infiltration.

### HOXA9-drug interactions

According to previous reports, in a few cancer types, *HOXA9* is known to induce resistance to therapeutically approved drugs, namely erlotinib, bevacizumab, and temozolomide [57, 58]. However, further studies are needed to determine the effectiveness of other antineoplastic drugs that target *HOXA9* in cancer treatment. According to the GDSC, *HOXA9* showed a positive correlation with drugs, namely SB52334, dabrafenib, PLX4720, YM155, and UNC0638, indicating that *HOXA9* could induce drug resistance in cancer. Notably, we found a negative correlation between *HOXA9* expression and linifanib, sorafenib, sunitinib, and midostaurin (Fig. 8C). This indicates that these drugs could increase the chemosensitivity of cells in elevated levels of *HOXA9*. Based on these observations, researchers should focus on utilizing these approved drugs to target *HOXA9* to restrain cancer progression.

## Discussion

Based on previous reports that suggested the role of *HOXA9* in human cancers, we systematically performed a pan-cancer analysis to determine the differential expression of *HOXA9*, its mode of regulation, its molecular mechanisms, and its functional implications using valid computational databases. Furthermore, its role as a biomarker for prognosis, stage stratification, correlation with immune cells of the tumor microenvironment, interactions with cofactors, and drug-gene interactions was also determined, aiming to understand the potential role of *HOXA9* in cancer progression.

Among the 33 TCGA cancer types analyzed using the RNA-Seq dataset, *HOXA9* was found to be highly upregulated in 9 cancer types and downregulated in 2 of the cancer types compared to normal tissues, indicating that *HOXA9* acts as an oncogene in the majority of cancer types. Apart from having prognostic significance, it indeed has clinical significance in differentiating the clinical stages of BRCA, HNSC, ESCA, KIRC, LIHC, LUSC, and STAD. These results suggest that *HOXA9* might serve as a diagnostic biomarker for these cancers (Table 2).

**Table 2:** *HOXA9* as a pan-cancer diagnostic indicator

S.No	Cancer type	Expression of HOXA9	Genetic alterations	Epigenetic alterations	Clinical significance
1	BRCA	Low↓	–	Hypermethylation	Differential expression in stages and immune subtypes
2	COAD	High↑	–	–	–
3	ESCA	High↑	Heterozygous amplification (65.21%)	–	Stages
4	HNSC	High↑	–	–	Stages
5	KIRP	High↑			Stages and immune subtypes, Immune infiltration
6	KIRC	Low↓	–	Hypermethylation	Stages
7	LIHC	High↑	–	–	Stages
8	LUSC	High↑	–	–	Stages and immune subtypes
9	PRAD	High↑	–	Hypomethylation	Stages and immune subtypes
10	READ	High↑	Heterozygous amplification (64.24%)	–	Stages
11	STAD	High↑	–	–	Stages and immune subtypes

Previous studies have shown that the aberrant expression of *HOXA9* in solid cancers primarily results from epigenetic factors. Our data reflect the minimal variation in genetic factors at the *HOXA9* locus; specifically, SNVs account for 32% of all cancers. Conversely, we observed a significant incidence of CNVs in READ (64.24%) and ESCA (65.21%). This emphasizes the necessity for focused research to validate and assess the impact of CNVs in these specific cancers. Researchers have demonstrated that *HOXA9* promoter methylation is responsible for its aberrant expression in lung adenocarcinoma (LUAD) [31], non-small cell lung cancer (NSCLC) [24, 25], high-grade noninvasive bladder cancer [29], and HNSC [59, 60]. Notably, research on cervical cancer (CC) revealed that the repression of *HOXA9* results from increased methylation of its first exon. Restoring its expression could mitigate cancer-associated biological processes [21]. The prognostic significance of *HOXA9* methylation has been well-studied in solid cancers [61]. Indeed, in our BRCA and KIRC datasets, the decreased expression of *HOXA9* may be linked to hypermethylation at the *HOXA9* promoter, while the increased *HOXA9* expression in PRAD is a result of promoter DNA hypomethylation. The usual correlation between promoter DNA methylation and gene repression does not always follow the conventional trend. Growing evidence has shown that promoter DNA methylation is also linked to gene activation in different biological contexts, including normal development and metastatic malignancies [52]. CpG promoter DNA methylation either facilitates the binding of enhancers to methylated DNA or blocks the binding of potential repressors to transcription start sites (TSSs), thereby inducing gene transcription [52]. Nevertheless, studies have demonstrated that transcription can also be governed by alternative promoters situated at considerable distances upstream from the TSS [52]. Currently, activation of HOX genes through DNA hypermethylation has been widely regarded as a novel epigenetic mechanism in cancer [62]. In this study, we observed a consistent relationship between promoter DNA hypermethylation and the upregulation of *HOXA9* in COAD, ESCA, HNSC, LIHC, and READ. This emphasizes the need for in-depth exploration of the molecular mechanisms involved to elucidate the regulatory pathways governing the transcriptional activation of genes.

In addition to promoter CpG methylation, *HOXA9* is regulated by miRNAs in NSCLC [26, 27], acute myeloid leukemia (AML) [63, 64], osteosarcoma [15], epithelial ovarian cancer [28], CRC [12], glioma [16, 65], and uveal melanoma [30]. The sponging of particular miRNAs to the HOXA9 3’UTR not only contributes to increased proliferation, migration, invasion, and metastasis but also facilitates tumor recurrence. Hence, it is worth studying the miRNA-mediated regulation of HOXA9 in different cancer types. In the present study, we identified 12 miRNAs, including two HOX cluster-embedded miRNAs, namely hsa-miR-196a-5p and hsa-miR-196b-5p, that correlate the expression of *HOXA9* across cancers. Validating these findings via in vitro studies might help researchers unravel the mode of regulation in several cancer types.

The HOXA9 transcription factor is frequently associated with its cofactors, namely MEIS1 and PBX, which are TALE homeodomain-containing cofactors, thereby enhancing its DNA binding ability [66, 67]. Similarly, we observed an association of HOXA9 with PBX1, PBX2, PBX3, and MEIS1 in all 10 cancer types except for STAD. As a transcription factor, it has an inherent ability to bind to target genes involved in various normal developmental processes [68–72] as well as during cancer progression [17–22, 73–76]. The tumor-promoting property of HOXA9 in EOC cells is mediated through the activation of *TGF-β2* [77]. Moreover, it induces an aggressive phenotype in OVC cells via transcriptional activation of its target gene, *CDH3* (P-cadherin) [78]. Studies have also determined the functional role of HOXA9 in the regulation of target genes, namely eNOs (endothelial nitric oxide synthase), *CDH5* (VE-cadherin), and *VEGFR2,* to promote angiogenesis [79]. Our analysis revealed the presence of 2247 targets of HOXA9, including angiogenic markers, EMT markers, and molecules of the Wnt/β-catenin, TGF-β, PI3K, NF-κB, and VEGF signaling pathways. Moreover, KEGG pathway analysis revealed the involvement of HOXA9 targets in the cell cycle, autophagy, metabolism, the AMPK pathway, Hippo signaling, PI3K/AKT signaling, TGF-β signaling, Rap1 signaling and transcriptional misregulation in cancer, indicating the potential role of those targets in cancer-associated biological processes.

According to our GO terms associated with HOXA9 targets, we determined that HOXA9 was localized mainly to the nucleus, cytoplasm, and microtubules. These cellular compartments control vital biological processes such as the cell cycle, actin filament organization, cell morphogenesis, and intracellular signal transduction. This regulation involves functions such as cadherin binding, GTPase regulator activity, protein serine-kinase activity, cell adhesion molecule binding, protein domain-specific binding, transcription factor binding, and transcription coregulator activity. Hence, additional studies are needed to assess the differential expression patterns of these targets in various cancers and validate the involved pathways.

Interestingly, the present study revealed that aberrant expression of *HOXA9* in KIRC and KIRP patients was associated with improved survival over time. This is due to the HOXA9-mediated activation of the antitumor immune response [53, 54]. Immune cells frequently interact with growing cancer cells in the microenvironment and play an influential role in tumor progression. To understand these dynamics, it is imperative to establish an association between the expression levels of various genes and immune infiltrating molecules. It has been reported that during the progression of prostate cancer (PCa), aberrantly expressed *HOXA2, HOXA9,* and *HOXA10* facilitate the infiltration of dendritic cells, macrophages, and mastocytes [80]. In EOC, HOXA9 facilitates the transcriptional activation of TGF-β2, which in turn triggers the efflux of chemokines such as CXCL12, IL-6, and VEGF-A in peritoneal fibroblasts [73, 81]. In our analysis, we observed variations in the expression of *HOXA9* in the immune subtypes BRCA, KIRP, LUSC, PRAD, and STAD. Elevated levels of *HOXA9* were significantly correlated with immune cell infiltration in most cancer subtypes, particularly in KIRP. This finding strengthens our previous observation that better survival in KIRP patients could be mainly due to the HOXA9-mediated induction of immune cell infiltration.

In summary, our study clarified the effect of *HOXA9* on the tumor immune response across various cancer types. Having elucidated the dynamics of cancer pathways, we identified drugs that target aberrantly expressed HOXA9. Our analysis of drug‒gene interactions revealed that drugs such as linifanib, sorafenib, sunitinib, and midostaurin have the potential to target *HOXA9* in cancer, thereby increasing chemosensitivity in cancer cells. Therefore, additional clinical trials are necessary in the future to assess the effectiveness of these drugs in combating aggressive phenotypes. Through our extensive computational analysis, we deduced that *HOXA9* serves as a potential biomarker across various cancers, laying the groundwork for further investigation into its role in specific cancer types. Despite conducting a thorough analysis, our study has a few limitations. The specific mechanism through which *HOXA9* contributes to oncogenicity in distinct cancer types is still not understood. There is a need to validate these findings through experimental work, both *in vitro* and in vivo, to elucidate the *HOXA9*-mediated dynamics of cancer progression. We recently identified the correlation between *HOXA9* and its targets, as well as its association with immune cells. More studies are needed to understand the exact pathways involved in cancer progression and immune infiltration. To target HOXA9 therapeutically, clinical trials for the newly proposed drugs must be carried out, considering the specific cancer types.

## Conclusion

This is the first comprehensive pan-cancer analysis of *HOXA9* to reveal its crucial role in human cancers. Through extensive computational analysis using experimentally validated datasets, we concluded that *HOXA9* could serve as a potential diagnostic biomarker for cancer detection and stage stratification. Moreover, our investigation revealed the impact of *HOXA9* on various signaling pathways and cancer hallmarks. Understanding the correlation between *HOXA9* expression and various immune cells has paved the way for clinicians to consider immunotherapy in various cancer types. Targeting *HOXA9* has emerged as a promising strategy with the potential to impede cancer progression, offering new perspectives for future research and clinical interventions in diverse cancer types.

## Supplementary Information

Below is the link to the electronic supplementary material.

Supplementary file1 (TIF 14903 KB)

Supplementary file2 (TIF 10414 KB)

Supplementary file3 (TIF 2475 KB)

Supplementary file4 (TIF 6546 KB)

Supplementary file5 (XLSX 12 KB)

Supplementary file6 (XLSX 255 KB)

Supplementary file7 (XLSX 38 KB)

Supplementary file8 (XLSX 340 KB)

## Data Availability

All data retrieved and analyzed in this study are included in the manuscript, and additional information has been provided as supplementary files.

## Abbreviations

GDC: Genomic Data Commons
TCGA: The Cancer Genome Atlas
ncRNA: Noncoding RNA
CRC: Colorectal cancer
PC: Pancreatic cancer
PCa: Prostate cancer
GBM: Glioblastoma
CSCC: Cutaneous squamous cell carcinoma
CC: Cervical cancer
BC: Breast cancer
NSCLC: Non-small cell lung cancer
EOC: Epithelial ovarian cancer
HGNOC: High-grade noninvasive bladder cancer
LUAD: Lung adenocarcinoma
HPA: Human protein atlas
IHC: Immunohistochemistry
CCLE: Cancer cell line encyclopedia
CNV: Copy number variations
SNV: Single-nucleotide variations
GSCA: Gene set cancer analysis
PPIN: Protein‒protein interaction network
ChIP: Chromatin immunoprecipitation
COAD: Colon adenocarcinoma
ESCA: Esophageal carcinoma
HNSC: Head and neck cancer
KIRP: Kidney renal papillary cell carcinoma
LIHC: Liver hepatocellular carcinoma
LUSC: Lung squamous cell carcinoma
PRAD: Prostate adenocarcinoma
READ: Rectal adenocarcinoma
STAD: Stomach adenocarcinoma
BRCA: Breast invasive carcinoma
KIRC: Kidney renal clear cell carcinoma

## Code availability

NA.
