# Supplementary figures and images for: Pan-cancer exploration of oncogenic and clinical impacts revealed that HOXA9 is a diagnostic indicator of tumorigenesis
Source: Clin Exp Med. 2024 Jun 21;24(1):134. doi: 10.1007/s10238-024-01389-x (PMC11192824; doi:10.1007/s10238-024-01389-x)

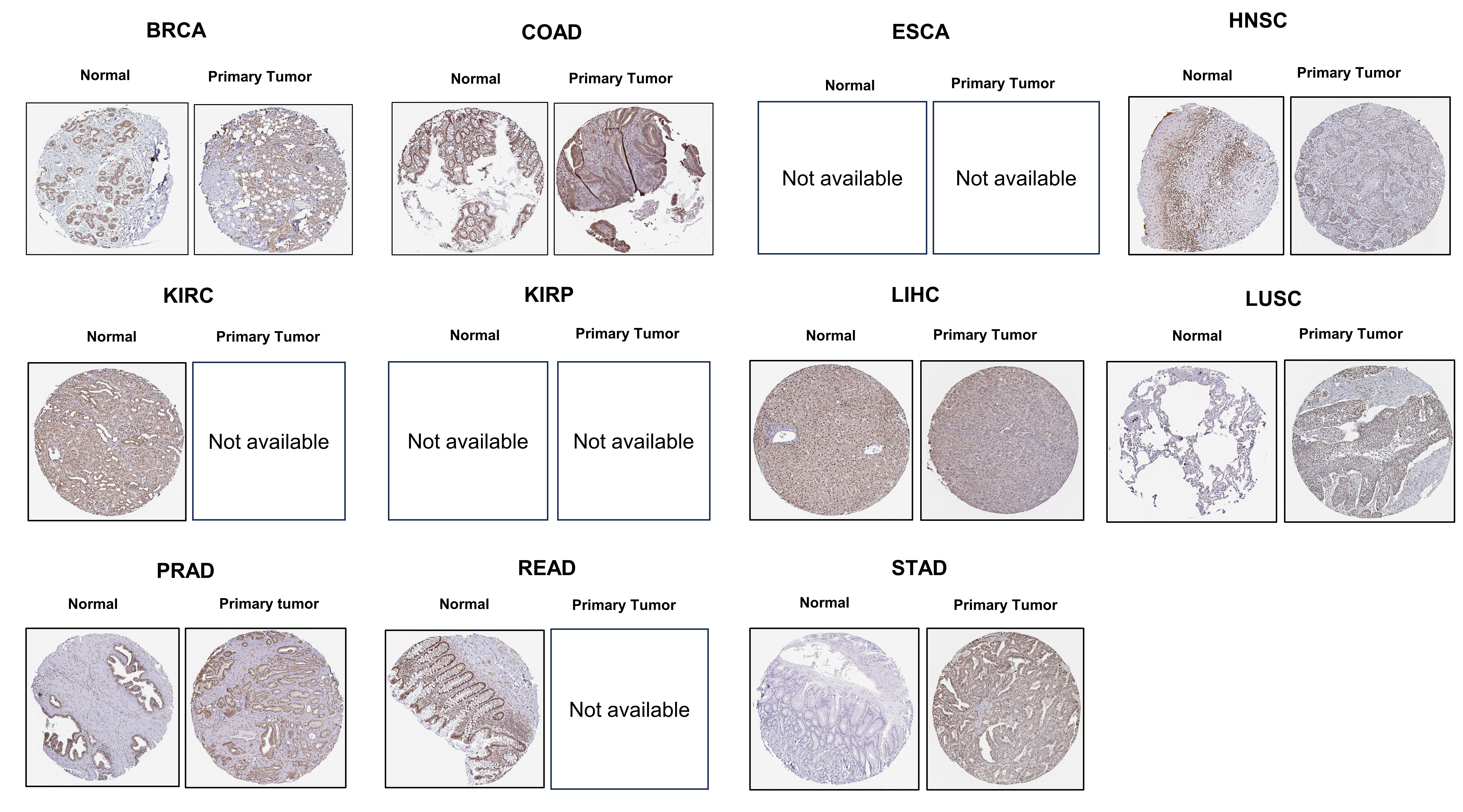

Supplement: Supplementary file 1 — Supplementary file1 (TIF 14903 KB) [file 10238_2024_1389_MOESM1_ESM.tif]

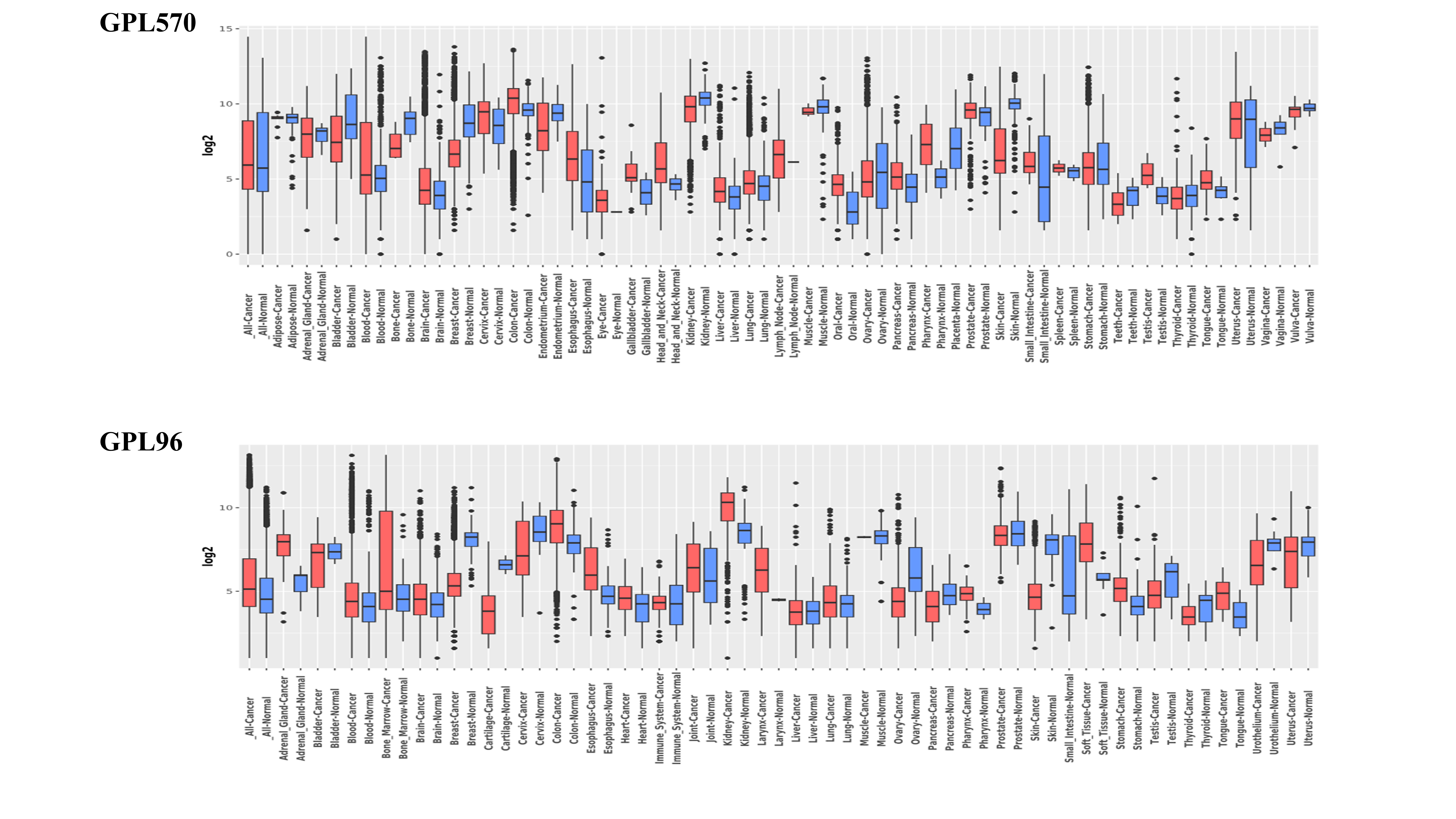

Supplement: Supplementary file 2 — Supplementary file2 (TIF 10414 KB) [file 10238_2024_1389_MOESM2_ESM.tif]

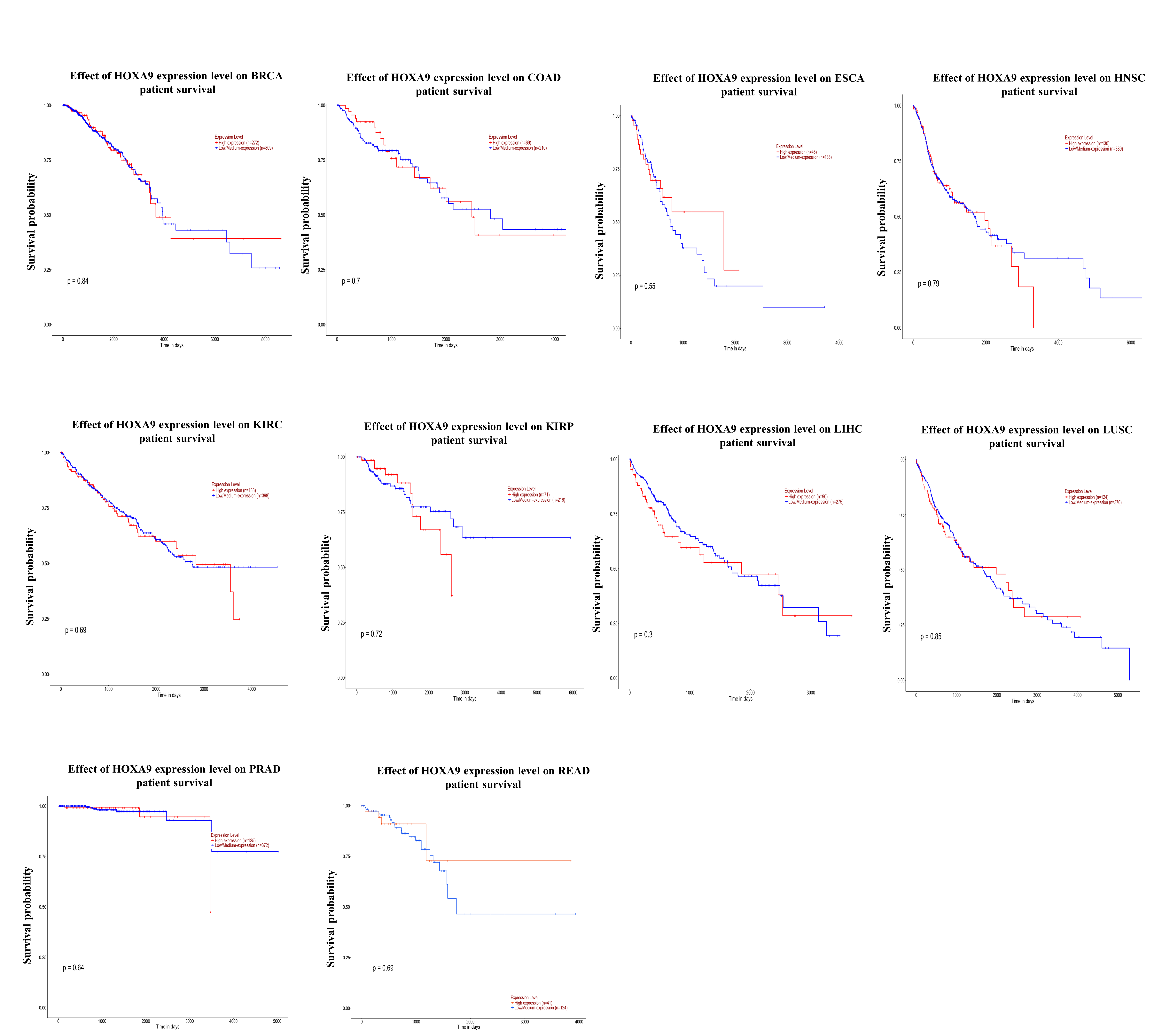

Supplement: Supplementary file 3 — Supplementary file3 (TIF 2475 KB) [file 10238_2024_1389_MOESM3_ESM.tif]

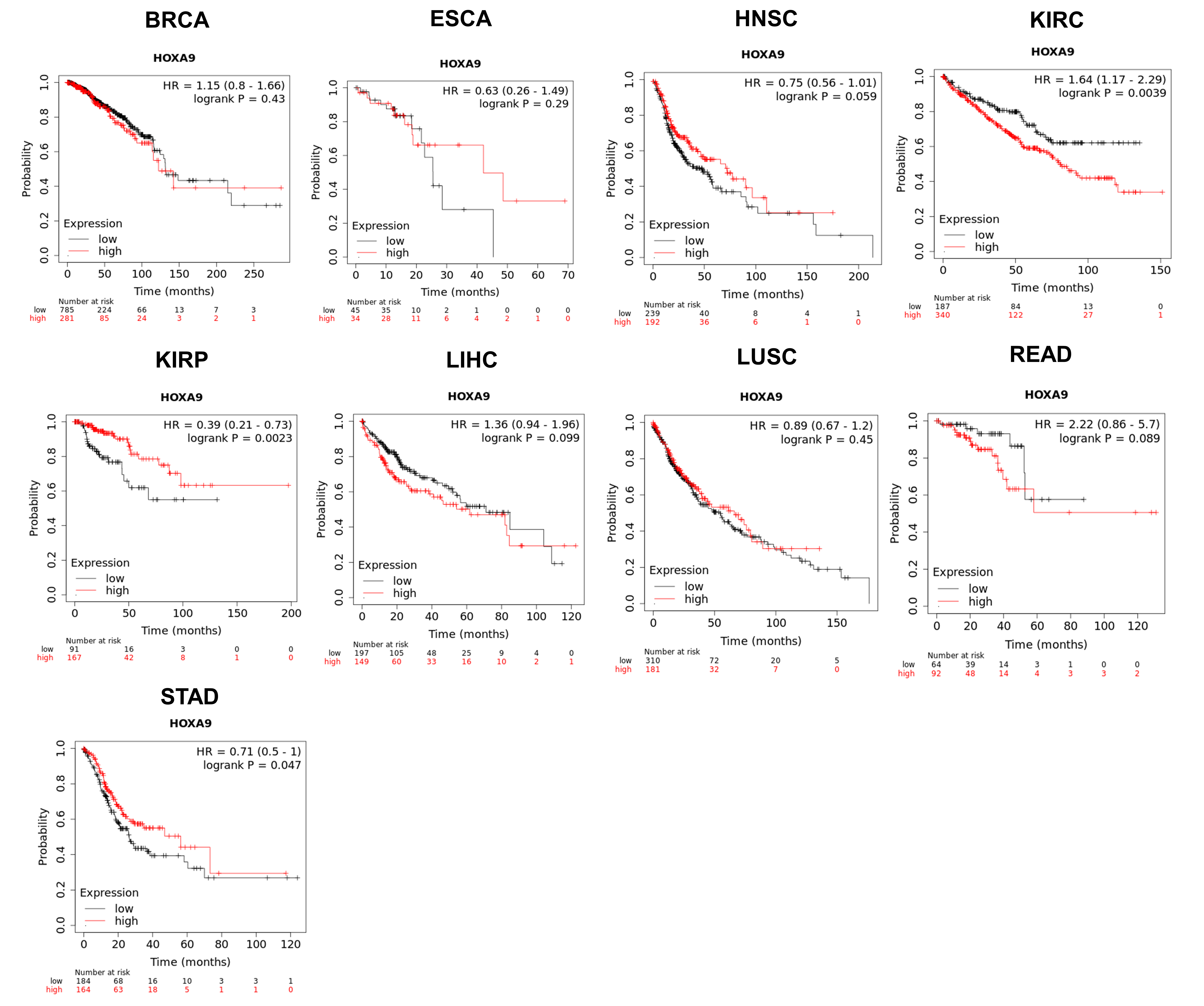

Supplement: Supplementary file 4 — Supplementary file4 (TIF 6546 KB) [file 10238_2024_1389_MOESM4_ESM.tif]
